# Effectiveness of ultraviolet–C robots in isolation room disinfection: Impact of dose, distance, and direct exposure on environmental surface decontamination

**DOI:** 10.1371/journal.pone.0359955

**Published:** 2026-11-04

**Authors:** Ying-Chun Chen, Wei-Ling Liao, Su-Chiu Wang, Chun-Hsi Tai

**Affiliations:** 1 Department of Nursing, Taichung Veterans General Hospital, Taichung, Taiwan; 2 Infection Control Center, Taichung Veterans General Hospital, Taichung, Taiwan; University of Pennsylvania, UNITED STATES OF AMERICA

## Abstract

**Background:**

To minimize environmental services staff’s exposure to pathogens, ultraviolet-C (UV-C) disinfection robots were applied before terminal manual environmental disinfection. This study aimed to evaluate the disinfection efficacy of the UV-C robot and to assess the effects of ultraviolet-C dose, distance, and direct exposure (line-of-sight irradiation) on disinfection outcomes.

**Methods:**

This before-after study was conducted in a negative-pressure isolation ward comprising 17 single-patient rooms at a medical center in Taiwan between August 24, 2024, and January 3, 2025, yielding a total of 270 paired samples. An ultraviolet-C-emitting robot with a wavelength of 254 nm was sequentially positioned on both sides of the patient bed and operated for 5 minutes on each side before routine manual environmental disinfection. Total aerobic colony counts (ACC) and bacterial culture positivity rates were obtained from high-touch environmental surfaces (e.g., bed rails, call bells and bedside chairs) before and after UV-C disinfection. The effects of UV-C dose, distance between the robot and surfaces, and the presence of direct exposure (line-of-sight irradiation) versus indirect exposure on disinfection efficacy were analyzed using multivariate binary logistic regression, performed using SPSS version 27.0. The results of the regression analysis were expressed as odds ratios (ORs) with 95% confidence intervals (CIs).

**Results:**

The median ACC and bacterial culture positivity rates on surfaces significantly decreased after UV-C disinfection (0.76 vs. 0.08 CFU/cm²; 83% vs. 57.4%, *P* < 0.001). A weak negative correlation was observed between ACC and UV-C doses after disinfection (*r*_s_ = −0.281, 95% CI: −0.390 to −0.164, *P* < 0.001), while no correlation (*r*_s_ = 0.040, 95% CI: −0.083 to 0.162, *P* = 0.510) and weak correlation (*r*_s_ = 0.317, 95% CI: 0.202 to 0.424, *P <*  0.001) were found between ACC at short and long irradiation distances. The ACC on environmental surfaces with direct UV-C exposure were significantly lower than those in shadowed areas [median: 0.00 (IQR: 0.00 to 0.44) vs. median: 0.16 (IQR: 0.00 to 0.80); *P* < 0.001]. Multivariate logistic regression identified direct exposure (line-of-sight irradiation) as the strongest predictor of negative bacterial culture results, reducing the odds of bacterial culture positivity by 79.5% compared to indirect exposure (OR = 0.205, 95% CI: 0.083 to 0.508, *P* < 0.001) in the layout of the negative pressure isolation room in this study.

**Conclusions:**

Ultraviolet-C robots serve as an effective disinfection intervention for hospital environments. Their efficacy is most pronounced when direct exposure (line-of-sight irradiation) is achieved in the isolation rooms. Therefore, aligning movable items toward the UV-C robot prior to disinfection, alongside subsequent manual environmental disinfection and physical debris removal, remains essential, particularly in shadowed areas.

## Introduction

Healthcare-associated infections (HAIs) impose a substantial economic burden on healthcare systems and remain a major threat to patient safety. Previous studies have reported that approximately 17% to 21.4% of environmental surfaces in rooms previously occupied by patients with healthcare-associated infections remained contaminated with common pathogens, including methicillin-resistant *Staphylococcus aureus* (MRSA), vancomycin-resistant *Enterococcus* (VRE), and *Clostridioides difficile*, even after terminal cleaning with disinfectant wipes [1–3]. Studies have shown that contaminated hospital surfaces remain a critical reservoir for multidrug-resistant pathogens despite standard terminal environmental disinfection, leading to rapid pathogen transfer and higher postoperative infection rates. This underscores the insufficiency of current protocols and the need for enhanced environmental disinfection [4,5].

Various strategies have been implemented to enhance hospital hygiene, including UV devices, hydrogen peroxide (HP) systems and antimicrobial coatings. The advantages of UV systems include shorter disinfection times and zero residue; however, their limitations include the inability to achieve physical cleaning, the requirement for room evacuation during operation, and an efficacy restricted to pathogens within direct or indirect lines of sight. For HP systems, the benefits comprise zero residue and a proven superior efficacy against spore-forming microorganisms; nevertheless, they require the sealing of ventilation systems during application and similarly fail to provide physical cleaning. In contrast, antimicrobial coatings face challenges regarding their antimicrobial spectrum, durability, disinfection efficiency, high costs, and maintenance [6,7].

The 2019 revision of the U.S. Centers for Disease Control and Prevention (CDC) Guideline for Environmental Infection Control in Health-Care Facilities emphasizes that adherence to standard operating procedures is essential for the thorough cleaning and disinfection of environmental surfaces and medical equipment, particularly high-touch areas. According to established guidelines, UV radiation is primarily recommended as a supplementary measure for air purification within airborne infection isolation rooms (AIIRs). Applications typically involve installing ultraviolet germicidal irradiation units within heating, ventilation, and air conditioning exhaust ducts to augment high-efficiency particulate air (HEPA) filtration or deploying upper-room UVGI fixtures to disinfect air near the ceiling [8]. However, these air-centric methods fail to achieve effective UV surface decontamination, leaving a critical gap that mobile UV disinfection robots are specifically engineered to fill. A growing body of evidence supports their utility in clinical settings; studies involving automated, portable, or fixed UV systems have demonstrated measurable reductions in multidrug-resistant organism (MDRO) colonization and infection rates, though the magnitude of these effects varies across the literature [9,10]. Compared to fixed overhead lamps or manual portable units, UV disinfection robots feature autonomous navigation, allowing them to reposition themselves in close proximity to target surfaces. This mobility circumvents shadowing effects and optimizes UV irradiance on complex environmental surfaces. In the wake of the COVID-19 pandemic, healthcare facilities have increasingly adopted these robotic systems to bolster environmental hygiene. They provide a contactless disinfection modality that reduces labor demands while limiting occupational exposure to infectious agents [11]. One study has also shown that during the COVID-19 pandemic, enhancing environmental disinfection with ultraviolet light reduced the contamination rates of environmental surfaces and healthcare workers’ personal protective equipment (PPE) from 48.4% and 13.3%, respectively, down to 0%, thereby mitigating the risk of occupational exposure for personnel [12].

Ultraviolet-C (UV-C), with a wavelength range of approximately 200–280 nm, is considered the most germicidal portion of the ultraviolet spectrum. Ultraviolet (UV) inactivates microorganisms by damaging nucleic acids and proteins, thereby inhibiting microbial replication [13,14]. However, the effectiveness of UV disinfection can be influenced by multiple factors, including the presence of organic matter, the UV dose, distance from the UV source, exposure time, and shielding caused by shadows or obstacles [15–18]. For actively growing bacteria, reported UV doses range from 0.4 to 6 mJ/cm² to achieve a 1 log_10_ (90%) reduction and from 1.1 to 14 mJ/cm² to achieve a 4 log_10_ (99.99%) reduction in bacterial counts for various pathogens [19].

The UV dose is equal to the UV intensity multiplied by the exposure time in seconds, where the UV intensity is inversely proportional to the square of the distance (measured in meters). Specifically, reducing the distance from 2 meters to 1 meter increases the UV intensity fourfold. Consequently, both shortening the distance and extending the exposure time can enhance the cumulative UV dose [17]. Furthermore, studies indicate that when UV sensors are placed at longer distances, on horizontal planes, and in shaded areas, the detected UV intensity is at its lowest, resulting in the smallest reduction in ACC [20].

In healthcare settings, UV devices are deployed in various functional configurations. For surface decontamination, devices are utilized sequentially around patient beds and operating tables to ensure multi-angle exposure, typically lasting five minutes per position, or installed as automated post-use systems in bathrooms [21–24]. Additionally, their application extends to air disinfection in clinics and corridors [25,26], as well as mobile disinfection at multiple locations within ambulances [27].

To minimize the exposure of environmental services personnel to airborne pathogens, a UV-C disinfection robot was deployed prior to standard manual environmental disinfection protocols following patient transfer or discharge. Unlike previous studies that predominantly focus on the combined efficacy of manual environmental disinfection and ultraviolet disinfection [22,23,28], this study specifically isolates and evaluates the standalone effectiveness of UV-C robotic disinfection. Furthermore, it assesses the impact of critical variables, including UV dose, distance from the source, and the influence of direct exposure (line-of-sight irradiation) versus indirect exposure. The findings of this study provide practical information to support environmental disinfection strategies and establish standardized procedures for environmental surface disinfection.

## Materials and methods

### Ethics statement

The study protocol was approved by the Institutional Review Board of Taichung Veterans General Hospital, which granted a Certification of Exemption from IRB Review (Approval No. CW23541B). This study focused exclusively on environmental surface decontamination and did not involve human participants, medical records, or biological samples. Because all environmental sampling was conducted in isolation rooms after patients had been transferred or discharged, the requirement for informed consent was waived.

### Study design and sampling sites

This before-and-after study was conducted in a negative-pressure isolation ward comprising 17 single-patient rooms at a tertiary medical center. The negative pressure isolation room measured 4 meters in length and 3.8 meters in width (Fig 1). Sampling sites included high-touch environmental surfaces, such as the bed rails, call bell, bedside table, bedside chair, suction machine, light switch, telephone, television (TV) remote control, and bathroom door handle. For each surface, the surface area was measured beforehand and divided into left and right sides; either the left or right side was randomly assigned to be sampled for bacterial culture before or after UV-C disinfection. Each sampling area measured approximately 10 × 10 cm; if the surface area was smaller, the entire surface was sampled.

**Fig 1:**
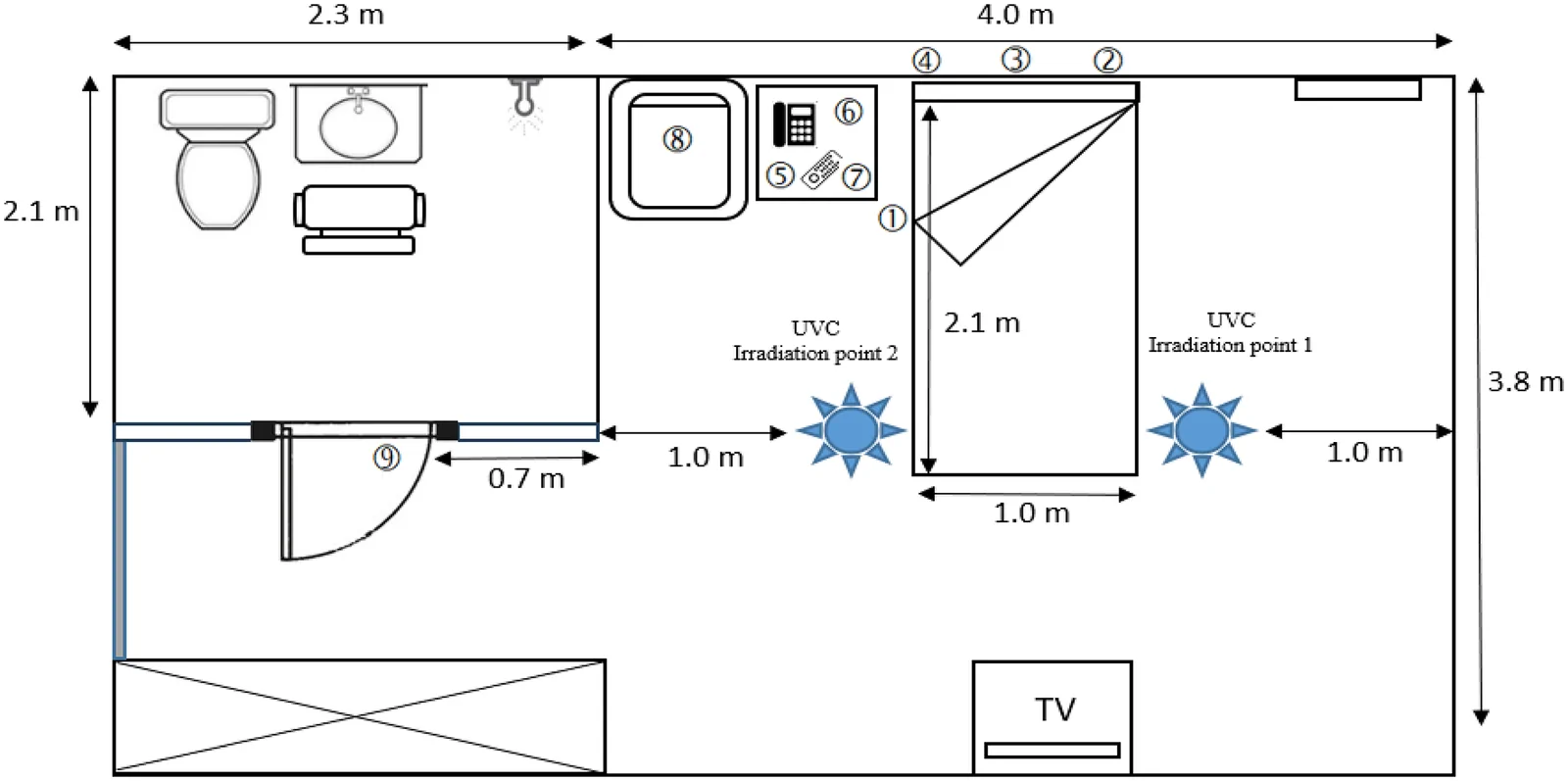
Typical isolation room layout: ① Bed rail; ② Calling bell; ③ Suction machine; ④ Light switch; ⑤ Bedside table; ⑥ Telephone; ⑦ TV remote control; ⑧ Bedside chair; ⑨ Bathroom door handle; 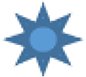 UV-C disinfection robot.

### Data collection

This study collected bacterial culture specimens, UV-C irradiation doses on environmental surfaces, the distance and the presence or absence of direct exposure between environmental surfaces and the UV-C disinfection robot.

After patients were transferred out of the isolation rooms or discharged, high-touch environmental surfaces were sampled for bacterial culture before UV-C disinfection. A sterile swab was moistened with thioglycollate broth (Creative Life Science Co., Ltd., New Taipei City, Taiwan). The swab was held at a 30^o^–45^o^ angle against the surface, and systematic horizontal and vertical strokes were performed while simultaneously rotating the swab shaft to maximize specimen recovery. Immediately following collection, each swab was placed into a test tube containing 8 mL of thioglycollate broth (as previously mentioned). Next, the UV intensity detector probe (Lutron LX-107 Light Meter, Lutron Electronic Enterprise Co., Taipei, Taiwan), which was calibrated prior to each use, was placed adjacent to the surface to be sampled after disinfection. A UV-C-emitting robot (Hyper Light Disinfection Robot; Mediland, Taoyuan, Taiwan) with a wavelength of 254 nm, using a high-output UV-C dose generated by an amalgam lamp and patented reflective cover technology, was sequentially positioned on both sides of the patient bed and operated for 5 minutes per side (Fig 1) [29]. Following UV-C exposure, the opposite side of each surface was sampled for bacterial culture. The ultraviolet-C dose was calculated as the measured average UV intensity multiplied by the total exposure time of 600 seconds (5 minutes on each side of the bed for a total of 10 minutes) [17]. The linear distances between each environmental surface and the UV robot during irradiation from both sides of the hospital bed were measured, and these distances were subsequently categorized into short and long distances for analysis. Furthermore, the exposure conditions were recorded based on the spatial alignment between the UV light source and the environmental surfaces: an exposure was defined as direct irradiation if the light source was perpendicular to the surface with an unobstructed line of sight; otherwise, it was categorized as indirect irradiation if the exposure was non-perpendicular or if physical obstructions were present.

### Laboratory method for total bacterial colony counting

For the colony counting of bacterial cultures, each swab sample was placed in a test tube containing 8 mL of thioglycollate broth. Within 30 minutes of collection, the specimens were sent to the laboratory. First, the thioglycollate broth containing the sampling swab was vortexed for 15 seconds. Next, a 1-mL aliquot of the solution was transferred via a sterile pipette onto a blood agar plate (an enriched medium, Creative Life Science Co., Ltd., New Taipei City, Taiwan). The diluted solution was then evenly spread across the plate using an L-shaped spreader and incubated at 37 °C for 48 hours in an incubator (Elite II, REVCO, Waltham, MA, USA). Subsequently, the ACC (expressed as colony-forming units, CFU) were determined with the assistance of a grid and a magnifying glass to reduce errors from manual interpretation and overlapping counting. The remaining samples were stored in a refrigerator at 4°C. For specimens with colony counts exceeding 300 CFU/plate, serial dilutions were performed up to a maximum of a 10^3^-fold dilution.

A negative control was not utilized in this study. In addition, the bacterial culture positivity rates before and after UV-C disinfection were calculated as the number of samples with positive bacterial growth divided by the total number of samples.

Data collection and specimen sampling for bacterial cultures were performed by a designated infection preventionist with over 15 years of experience, adhering strictly to standard operating procedures. Laboratory procedures for bacterial culture were carried out by a separate medical technologist, who was blinded to the timing of specimen collection (i.e., before or after UV-C irradiation).

### Sample size and sampling strategy

Environmental sampling was conducted across 17 negative pressure isolation rooms, targeting nine designated surfaces per room. To ensure that the results for each surface type were sufficiently representative, specimens were sequentially collected from rooms occupied by 30 unique patients enrolled over the study period. For each sampling event associated with a specific patient’s stay, one sample before the intervention and one sample after the intervention were obtained from each of the nine designated surfaces. Because the facility contained only 17 available isolation rooms, certain rooms were sampled repeatedly at different time points (corresponding to the occupancy of different unique patients) to achieve the target sample size.

The sample size was justified using a power analysis performed with G*Power 3.1.9.7. For the Wilcoxon signed-rank test (used to compare ACC before and after disinfection), a minimum of 47 pairs was required to detect a medium effect size (*dz* = 0.5) with a significance level (α) of 0.05 and a power (1– β) of 0.90. A minimum total sample size of 187 was determined to be sufficient for a multivariable logistic regression. This calculation was based on a two-tailed Wald’s Z test, an anticipated odds ratio (OR) of 0.186 (based on the actual statistical data), a baseline probability of 0.83 (based on the actual statistical data), an alpha level of 0.05, a statistical power of 0.90, and a R2 of 0.589 (based on the calculations of the actual statistical data using Linear Regression) allocated for other controlling variables (distance and energy). However, to ensure the representativeness and ecological validity of the findings across various physical environments, nine distinct environmental surfaces were evaluated, with 30 independent samples collected from each surface during both the before- and after-intervention phases (totaling 60 samples per surface and yielding a final total of 540 samples). This deliberate oversampling was chosen to mitigate sampling bias tied to surface-specific material properties and to strengthen the statistical power for evaluating the three primary predictors (distance, energy, and direct exposure)**.**

### Statistical analysis

All statistical analyses were performed using SPSS version 27.0 (IBM Corp., Armonk, NY, USA). The bacterial density on environmental surfaces was calculated using the following verbal formula: the number of colony-forming units (CFUs) counted on the agar plate was multiplied by the corresponding dilution factor and the total volume of the initial thioglycollate broth (8 mL), and then divided by the product of the inoculation volume plated (mL) and the total surface area sampled (cm^2^).

Differences in ACC before and after UV-C disinfection were assessed using the Wilcoxon signed-rank test. This non-parametric method was selected because the ACC data were not normally distributed; therefore, medians were used for comparison. The McNemar’s test was used to compare the positivity rates of bacterial cultures before and after UV disinfection. Spearman rank correlation was used to assess the associations between UV-C dose and ACC, and between distance and ACC after disinfection. The Mann–Whitney U test was employed to assess differences in the presence or absence of direct exposure. The strength of the associations between UV dose, distance, direct exposure, and surface culture results after disinfection was evaluated using multivariate binary logistic regression. Statistical significance was defined as *P* < 0.05.

## Results

Between August 24, 2024, and January 3, 2025, a total of 270 paired environmental surface samples (n = 540; before and after disinfection) were collected from 17 single-patient rooms in this study.

### Effect of UV-C disinfection on ACC and culture positivity rates

For 270 environmental surfaces, the range, median (interquartile range, IQR) of ACC were 0.00 to 178.82, 0.76 (0.11 to 3.44) CFU/cm² before UV-C disinfection and 0.00 to 20.71, 0.08 (0.00 to 0.73) CFU/cm² after disinfection, respectively (*P* < 0.001) (Table 1). The intervention led to a substantial reduction in the median microbial burden (89.5%), with 42.6% of the samples showing complete decontamination (0 CFU) after disinfection. Higher median CFU values were observed on the telephone and TV remote control after disinfection. The bacterial culture positivity rate decreased significantly from 83% (224/270) before disinfection to 57.4% (155/270) after disinfection (*P* < 0.001).

**Table 1:** Total aerobic colony counts (CFU/cm²) on environmental surfaces before and after UV-C disinfection.

Item	n	Before UV-C disinfection		After UV-C disinfection		*P* value *
		Median	IQR	Median	IQR	
Bed rail	30	0.16	0.08-0.86	0.00	0.00-0.08	< 0.001
Call bell	30	0.73	0.00-4.36	0.00	0.00-0.00	0.002
Bedside table	30	1.04	0.40-7.96	0.16	0.00-1.44	< 0.001
Bedside chair	30	1.24	0.24-4.44	0.08	0.00-0.56	< 0.001
Suction machine	30	0.28	0.08-1.82	0.08	0.00-0.32	0.001
Light switch	30	0.75	0.00-2.45	0.00	0.00-0.75	0.011
Telephone	30	2.08	0.54-8.54	0.44	0.08-1.62	< 0.001
TV remote control	30	2.71	0.68-11.27	0.82	0.12-2.80	0.002
Bathroom door handle	30	0.39	0.22-2.26	0.17	0.08-0.44	0.004
Total	270	0.76	0.11-3.44	0.08	0.00-0.73	< 0.001

* P values were calculated using the Wilcoxon signed-rank test for paired comparisons before and after UV-C disinfection.

### Effect of UV-C doses on ACC after disinfection

Among the 270 environmental surfaces sampled after UV-C disinfection, the measured UV-C doses ranged from 0 to 906 mJ/cm², with a mean of 125.87 mJ/cm² (±172.28) and a median of 19.80 (IQR: 6.00 to 258.15). Of these, 51 surfaces (18.89%) received no UV-C exposure (0 mJ/cm^2^), which fell within the lowest dose range (0.00 to 6.0mJ/cm^2^) due to being located in blind spots or shadowed areas. These unexposed surfaces included 6 bed rails, 9 bedside tables, 1 bedside chair, 7 suction machines, 8 telephones, 8 television remote controls, and 12 bathroom door handles, all of which were not fully exposed to UV-C irradiation. The remaining 219 surfaces (81.11%) received UV-C doses ranging from 6 to 906 mJ/cm², all of which exceeded the energy threshold of 1.1–14 mJ/cm² required to achieve a 4-log_10_ reduction in bacterial load. A weak negative correlation was observed between ACC and UV-C doses after disinfection (*r*_s_ = –0.281, 95% CI: −0.390 to −0.164, *P* < 0.001) (Fig 2) (Table 2).

**Table 2:** UV-C doses (mJ/cm²) on environmental surfaces during UV-C irradiation.

Item	n	Mean ± SD	Median	IQR	Min – Max
Bed rail	30	62.10 ± 118.72	42.00	6.00-76.20	0-654
Call bell	30	353.60 ± 87.05	330.00	298.50-373.50	216-540
Bedside table	30	4.40 ± 3.12	6.00	0.00-6.00	0-12
Bedside chair	30	153.54 ± 116.60	141.00	49.50-253.05	0-456
Suction machine	30	32.60 ± 31.26	24.00	4.50-66.00	0-108
Light switch	30	344.20 ± 116.87	339.00	282.00-382.50	150-540
Telephone	30	4.80 ± 3.31	6.00	0.00-6.00	0-12
TV remote control	30	4.60 ± 3.02	6.00	0.00-6.00	0-12
Bathroom door handle	30	173.02 ± 245.91	32.10	0.00-288.00	0-906
**Total**	**270**	**125.87 ± 172.28**	**19.80**	**6.00-258.15**	**0-906**

**Fig 2:**
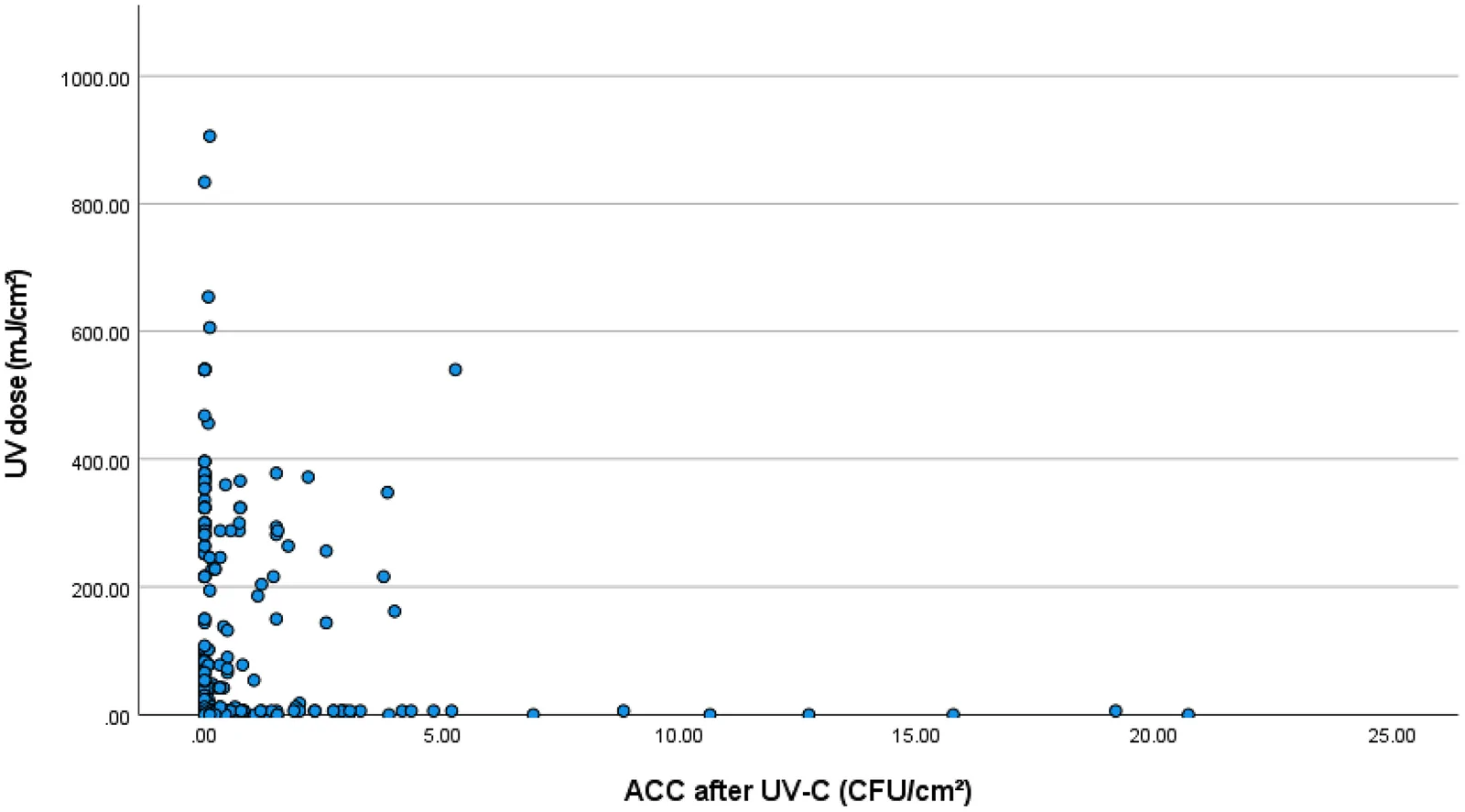
Scatter plot of UV dose vs. ACC after UV-C disinfection.

### Effect of exposure distance on ACC after UV-C disinfection

The UV-C robot performed disinfection on both sides of the patient bed. The distances from the UV-C robot to the environmental surfaces on both sides were measured and categorized into short and long irradiation distances to conduct correlation analyses with ACC after disinfection. There was no significant correlation between ACC and the short irradiation distance after disinfection (*r*_s_ = 0.040, 95% CI: −0.083 to 0.162, *P* = 0.510) (Fig 3A). Long irradiation distances showed a weak positive correlation with ACC after disinfection (*r*_s_ = 0.317, 95% CI: 0.202 to 0.424, *P* < 0.001) (Fig 3B) (Table 3).

**Table 3:** Correlation between distance (cm) and ACC.

Item	n	Mean ± SD	Median	IQR	Min – Max	Correlation
Short distance	270	171.46 ± 28.72	178.00	154.75-190.00	93-226	*r*_s_ = 0.040, 95% CI: −0.083–0.162, *P* = 0.510
Long distance	270	230.42 ± 54.96	220.00	198.75-246.25	128-389	*r*_s_ = 0.317, 95% CI: 0.202–0.424, *P <* 0.001

**Fig 3:**
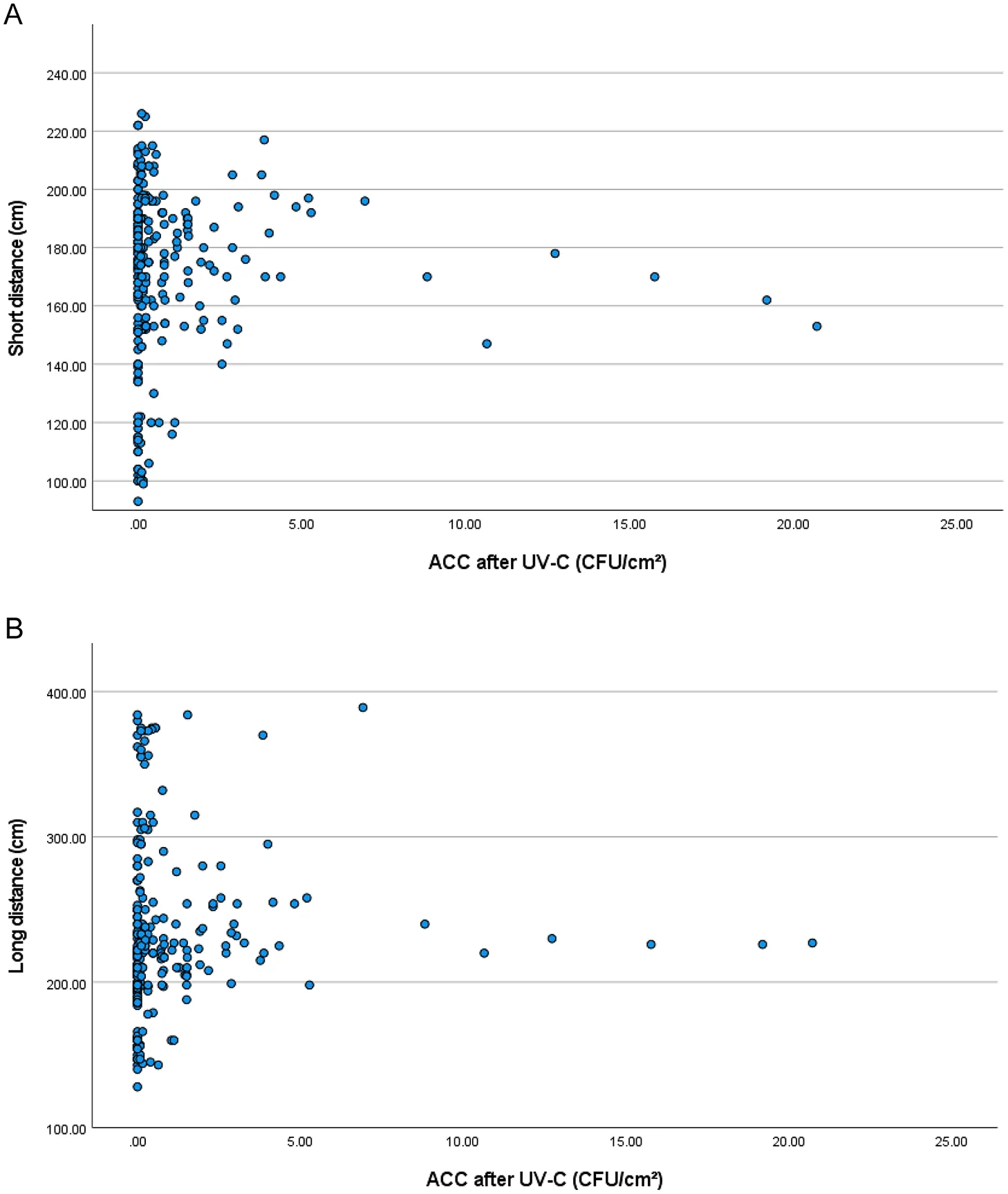
Scatter plot of UV-C distance vs. ACC after disinfection. (A) Scatter plot of short irradiation distance vs. ACC after disinfection. (B) Scatter plot of long irradiation distance vs. ACC after disinfection.

### Effects of direct versus indirect ultraviolet exposure on ACC after UV-C disinfection

Of the 270 environmental surfaces, 95 surfaces (35.2%) were positioned in direct exposure (line-of-sight of the irradiation source) median of 0.00 (IQR: 0.00 to 0.44) CFU/cm², while 175 surfaces (64.8%) were in shadow areas (indirect exposure) with a median of 0.16 (IQR: 0.00 to 0.80) CFU/cm². Total aerobic colony counts were significantly lower on surfaces that received direct UV-C exposure compared to those that did not (*P* < 0.001) (Table 4).

**Table 4:** Total aerobic colony counts (CFU/cm²) on environmental surfaces between direct versus indirect exposure.

Item	Direct exposure			Indirect exposure			*P* value *
	n	Median	IQR	n	Median	IQR	
Bed rail	0	–	–	30	0	0.00-0.08	
Call bell	30	0	0−0	0	–	–	
Bedside table	0	–	–	30	0.16	0.00-1.44	
Bedside chair	23	0.08	0.00-0.48	7	0.24	0.00-0.80	
Suction machine	0	–	–	30	0.08	0.00-0.32	
Light switch	30	0	0.00-0.75	0	–	–	
Telephone	0	–	–	30	0.44	0.08-1.62	
TV remote control	0	–	–	30	0.82	0.12-2.80	
Bathroom door handle	12	0.28	0.11-0.52	18	0.11	0.00-0.36	
**Total**	**95**	**0.00**	**0.00-0.44**	**175**	**0.16**	**0.00-0.80**	< 0.001

* P values were calculated using the Mann–Whitney U test.

### Multivariate binary logistic regression analysis of the effect of UV-C robot disinfection on bacterial culture positivity

There were 4 predictors included in the multivariate logistic regression model, which consisted of UV-C dose, distance (short and long irradiation distances), and direct/indirect irradiation. Irradiation distance was analyzed as a continuous variable measured in centimeters. The total number of outcome events (positive cultures) was 155 after disinfection.The results of the multivariate binary logistic regression analysis were summarized in Table 5. Long irradiation distance (OR = 0.986 per cm increase, 95% CI: 0.980 to 0.993, *P <* 0.001) and direct exposure (OR = 0.205, 95% CI: 0.083 to 0.508, *P <* 0.001) were significant independent predictors of bacterial culture results. However, no significant association was observed for short irradiation distance (OR = 0.996 per cm increase, 95% CI: 0.985 to 1.007, *P* = 0.467) and UV-C dose (OR = 1.001, 95% CI: 0.999 to 1.003, *P* = 0.467) (Table 5).

**Table 5:** Multivariate binary logistic regression analysis of exposure factors (UV-C dose, distance, and direct exposure) affecting bacterial culture results.

Variable	Bacterial culture results (negative vs. positive)		
	Odds Ratio	95% CI	*P* value
UV-C dose (mJ/cm²)	1.001	0.999-1.003	0.467
Short irradiation distance (cm)	0.996	0.985-1.007	0.467
Long irradiation distance (cm)	0.986	0.980-0.993	< 0.001
Direct exposure	0.205	0.083-0.508	< 0.001

## Discussion

This study investigated the disinfection efficacy of UV-C robots as a standalone intervention in negative-pressure isolation rooms, as well as the impact of UV-C dose, distance, and line-of-sight exposure. The results demonstrated that UV-C robots significantly reduced microbial loads and bacterial culture positivity rates even before manual environmental disinfection. A significant but weak negative correlation was observed between UV-C dose and ACC after disinfection, whereas long irradiation distance showed significant but weak positive correlations with the ACC. Within the specific spatial layout of the negative-pressure rooms in this study, direct exposure was identified as the most critical predictor influencing the disinfection efficacy of UV-C robots.

Previous studies comparing manual environmental disinfection alone to manual environmental disinfection combined with UV-C disinfection have shown that surfaces subjected to manual environmental disinfection alone maintain bacterial culture positivity rates ranging from 8.8% to 64.3%. This wide variation may be attributed to differing levels of surface contamination and diverse manual environmental disinfection protocols, including the use of various chemical disinfectants. In contrast, the integration of UV disinfection following manual environmental disinfection can substantially reduce the bacterial culture positivity rate to between 0% and 17.5% [22,23,28,30]. A distinctive feature of the present study is the immediate application of UV-C disinfection following patient discharge or transfer, assessing the impact of UV-C irradiation alone without prior manual environmental disinfection. Although both the ACC and the culture positivity rate decreased significantly, the latter remained high at 57.4%. This study differs from the aforementioned literatures in terms of study setting, UV robot brand, irradiation angle, exposure duration, and outcome measures. Consequently, a direct comparison regarding whether its efficacy is superior to manual environmental disinfection cannot be established. Nevertheless, our findings demonstrate that environmental disinfection of negative-pressure isolation rooms using the UV robot specified in this study can significantly reduce both ACC and culture positivity rates.

### Dose effects

A weak negative correlation between UV-C dose and disinfection efficacy appeared to contradict physical principles. This discrepancy likely stems from the fact that 80.11% of the environmental surfaces received a UV-C dose sufficient to achieve a 4-log_10_ reduction, leading to massive bacterial eradication. The computational logic involves first ranking the raw data in ascending order and subsequently calculating the correlation based on these ranks. Ideally, a perfect negative correlation is achieved when an increase in the UV dose (greater energy delivery) corresponds precisely to a reduction in the ACC [20,31]. In reality, however, when a substantial proportion of environmental surfaces yield a colony count (Y) of zero (42.6%, 115/270) in this study, the presence of these extensive tied ranks implies that the rank difference (*d*_*i*_) in the formula depends solely on the unidirectional variation in the ranks of X (dose). Mathematically, this phenomenon directly leads to a severe attenuation of the correlation coefficient toward zero [32].

This explanation was associated with the significant decrease in the ACC, with the median (IQR) dropping from 0.76 (0.11 to 3.44) CFU/cm^2^ before disinfection to 0.08 (0.00 to 0.73) CFU/cm^2^ after disinfection, representing an 89.5% reduction**.** Results from the multivariate binary logistic regression also indicated that the UV-C dose was not a significant predictor of bacterial culture positivity. This may be attributed to the fact that other confounding factors, such as shadowing, significantly affect the actual UV-C dose received by environmental surfaces [15,20].

### Distance effects

Theoretically, an increase in distance reduces the UV dose received by environmental surfaces, thereby leading to a increase in ACC [17,20,31]. Previous studies exploring the relationship between distance and disinfection efficacy typically employed a fixed-point irradiation design, which established a definitive distance between the UV-C source and the target surfaces [20,21,31,33]. In contrast, this study utilized a UV-C robot that performed disinfection for 5 minutes on each side of the patient bed. Consequently, environmental surfaces received varying degrees of radiant energy from both sides, which may have weakened the explanatory power of distance on disinfection efficacy, thereby resulting in a low correlation between distance and disinfection efficacy. This also accounts for the seemingly paradoxical finding in the multivariate binary logistic regression analysis, which indicated that each 1-cm increase in distance was associated with a 1.4% reduction in the odds of a positive bacterial culture (OR = 0.986; 95% CI: 0.980 to 0.993; *P* < 0.001). This contradicts previous findings that increasing distance diminishes radiant energy, thereby reducing disinfection efficacy [17,20]. Multi-angle irradiation might limit the predictability of bacterial cultures in this study after disinfection. However, 89.3% (241/270) of the environmental surfaces in the negative-pressure isolation rooms fell within the UV-C robot’s recommended 2-meter effective range. Therefore, most surfaces still received an irradiation dose capable of inducing a 4-log_10_ reduction in bacterial load.

### Direct exposure effects

Applying the UV-C robot to the layout of the negative-pressure isolation rooms in this study suggested that direct UV exposure was the only significant predictor of disinfection efficacy; specifically, direct exposure on environmental surfaces might be attributable to reduce the odds of a positive bacterial culture by 79.5%. This aligns with the study design by Boyce et al. (2016), which clearly demonstrated that a 90-degree perpendicular angle (direct exposure) yielded the highest efficacy in terms of increasing radiant energy and reducing bacterial loads, followed by a 0-degree horizontal angle (direct exposure), whereas a 0-degree horizontal angle with indirect exposure performed the worst [20]. These findings can be translated into a straightforward operational protocol: cleaning staff should be educated to orient movable environmental items toward the UV robot prior to irradiation. For immovable surfaces, strategies such as multiple irradiations from different angles or configuring the robot to navigate autonomous paths should be implemented to ensure direct UV exposure on all environmental surfaces, thereby achieving optimal disinfection outcomes.

### Limitations

This study has several limitations. First, the UV-C exposure was administered from two angles, making it difficult to define and analyze a single fixed irradiation distance. Second, although the UV-C disinfection robot used in this study was equipped with patented reflective cover technology; it was challenging to evaluate the isolated impact of this feature on the outcomes because not all environmental surfaces received direct UV-C irradiation. Third, since ACC were used rather than identifying specific pathogens, variations in the intrinsic resistance of the local flora across diverse organism species could have influenced the results. Fourth, this study evaluated the standalone efficacy of UV-C disinfection prior to manual environmental disinfection. This reversed sequence was intentionally designed to prioritize the occupational safety of environmental services staff by minimizing their exposure to high initial bioburden inside negative-pressure isolation rooms. However, we acknowledge this clinical adaptation as a methodological limitation. The presence of baseline organic matter, soils, and potential biofilms can act as physical barriers that shield underlying microorganisms, which may have led to an underestimation of the maximum germicidal capacity of the UV-C robot. Furthermore, because UV-C irradiation can induce protein denaturation and chemical cross-linking, this sequence could theoretically enhance the adherence of remaining organic stains to environmental surfaces. However, evaluating whether this altered workflow physically hardens real-world stains or increases the difficulty of subsequent manual scrubbing was beyond the clinical scope of the current study. Future research is warranted to comprehensively investigate the long-term operational trade-offs between healthcare worker safety and physical soil clearance efficiency. Additionally, future investigations should expand on these findings by comprehensively evaluating the environmental disinfection efficacy across three distinct operational modalities: pre- and post-intervention of standalone automated UV robot disinfection, manual environmental disinfection alone, and a combined approach integrating both manual environmental disinfection and UV disinfection. Fifth, due to practical personnel constraints within the Infection Control Center laboratory, microbiological plating and colony counting were conducted by a single dedicated medical technologist. Although this single-operator protocol eliminates inter-observer variability, future studies incorporating dual-operator independent counting would further strengthen data validation. Sixth, A major limitation of this study is that specimen processing was conducted under standard laboratory conditions without a classified cleanroom environment (e.g., Class 100/10,000) or parallel negative control groups. Therefore, the risk of minor exogenous contamination during manual handling cannot be entirely eliminated, and the 57.4% culture positivity rate should be interpreted cautiously as a conservative estimate of microbial clearance. Finally, dedicated chemical neutralizers were not added to the sampling broth. Although sampling was executed prior to terminal chemical disinfection and high broth volume dilution minimized active carryover, potential interference from trace surface disinfectant residues cannot be completely excluded.

## Conclusion

This study demonstrates that automated UV-C disinfection robots serve as an effective technological adjunct to enhance hospital infection control by significantly reducing the bacterial burden on environmental surfaces within negative-pressure isolation rooms. However, given that nearly 60% of surfaces still exhibited bacterial growth after the intervention, these systems must be integrated into a multimodal environmental disinfection protocol rather than utilized as a standalone solution. Multivariate analysis confirmed that direct line-of-sight UV-C exposure is a significant independent predictor of achieving negative bacterial culture outcomes. To translate these findings into clinical practice, hospital infection control committees should establish standardized workflows. Prior to device activation, movable clinical items should be systematically positioned as close to the robot as possible and oriented directly toward the radiant light source. Concurrently, for immovable fixtures, multi-angle irradiation positioning must be enforced to mitigate geometric shadow zones. Based on the parameters tested in this study, an exposure time of at least 5 minutes of direct, line-of-sight exposure per position is recommended. Finally, items frequently located in shadowed zones or beyond the effective radiation range—such as bed rails, telephones, TV remote controls, and bathroom door handles—must receive targeted, supplementary manual disinfection to ensure comprehensive decontamination.

## Supporting information

S1 Data List of ACC before and after UV-C disinfection, bacterial culture results, UV-C doses, distances, and direct exposure statuses for each environmental surface. (XLSX)
